# Surfactant protein D, Club cell protein 16, Pulmonary and activation-regulated chemokine, C-reactive protein, and Fibrinogen biomarker variation in chronic obstructive lung disease

**DOI:** 10.1186/s12931-014-0147-5

**Published:** 2014-11-25

**Authors:** Sofie Lock-Johansson, Jørgen Vestbo, Grith Lykke Sorensen

**Affiliations:** Institute of Molecular Medicine, University of Southern Denmark, JB Winsloews Vej 25.3, Odense, 5000 Denmark; Department of Respiratory Medicine, Gentofte Hospital, Hellerup, Denmark; Respiratory Research Group, Manchester Academic Science Centre University Hospital South Manchester NHS Foundation Trust Manchester, Manchester, UK

**Keywords:** COPD, Biomarker, SP-D, CC-16, PARC/CCL-18, CRP, Fibrinogen

## Abstract

Chronic obstructive pulmonary disease (COPD) is a multifaceted condition that cannot be fully described by the severity of airway obstruction. The limitations of spirometry and clinical history have prompted researchers to investigate a multitude of surrogate biomarkers of disease for the assessment of patients, prediction of risk, and guidance of treatment. The aim of this review is to provide a comprehensive summary of observations for a selection of recently investigated pulmonary inflammatory biomarkers (Surfactant protein D (SP-D), Club cell protein 16 (CC-16), and Pulmonary and activation-regulated chemokine (PARC/CCL-18)) and systemic inflammatory biomarkers (C-reactive protein (CRP) and fibrinogen) with COPD. The relevance of these biomarkers for COPD is discussed in terms of their biological plausibility, their independent association to disease and hard clinical outcomes, their modification by interventions, and whether changes in clinical outcomes are reflected by changes in the biomarker.

## Introduction

Chronic obstructive pulmonary disease (COPD) is a common disease worldwide [1] and is forecasted to be the 3rd leading cause of death globally in 2030 [2]. There is an unmet need for easily obtained biomarkers that can identify subtypes of COPD, predict outcomes of COPD, and that can evaluate and facilitate targeting in management of COPD. To aid in the evaluation and development of biomarkers for COPD, Sin & Vestbo have formulated 5 criteria as an extension from the criteria for a biomarker proposed by Bucher et al. [3]. These criteria are: biological plausibility, independent association to disease, hard clinical outcomes, modification by interventions, and whether changes in clinical outcomes are reflected by changes in the biomarker [4]. The aim of this review is to summarize observations for a selection of investigated biomarkers for COPD, with guidance from these criteria. The selected biomarkers are recently validated with regards to sensitivity, accuracy, precision and reproducibility [5]. Surfactant protein D (SP-D), club cell protein 16 (CC-16, previously named Clara cell protein), and pulmonary and activation-regulated chemokine (PARC/CCL-18) are categorized as pulmonary inflammatory markers, while C-reactive protein (CRP) and fibrinogen are systemic inflammatory markers.

### SP-D

SP-D is a member of the collectin family [6] and is primarily produced in type II pneumocytes [7-9].

### Biological plausibility

SP-D plays an essential role in pulmonary innate immune defense [10] and SP-D deficient mice exhibit abnormal accumulation of apoptotic macrophages. These animals also exhibit increased cytokine activation accompanied by lymphocyte infiltration and emphysema development [11-15], suggesting a protective role for SP-D in the pathogenesis of COPD. Several single nucleotide polymorphisms (SNPs) in the SP-D gene (*Sftpd*) have shown to associate with serum SP-D levels. In a twin-study, the coding SNP rs721917 was associated with lower SP-D levels in serum [16]. This finding was replicated in a cohort consisting of 1,719 COPD subjects in the “Evaluation of COPD Longitudinally to Identify Predictive Surrogate Endpoints” Study (ECLIPSE), where 28 SNPs were shown to influence serum SP-D. However, the strongest effect occurred with a promotor SNP (rs1885551), where the minor allele associated with lower serum SP-D [17]. In addition, three *Sftpd* SNPs (including rs721917) have been associated with forced expiratory volume (FEV_1_)% predicted [17], and five SNPs in *Sftpd* have been associated with an increased risk of COPD development in a Genome Wide Association study (GWAS) [18]. Lastly, a Japanese study reported one *Sftpd* SNP in association with emphysema [19].

### Independent association to disease

SP-D has been measured in bronchoalveolar lavage fluid (BALF) and in blood. Among smokers, most studies have reported reduced levels of SP-D in BALF [20-23] and increased circulating levels [16,22,24,25]. Some studies have noted significant decreases in SP-D in BALF from COPD patients compared with current smokers [22] while others have not [20,21]. SP-D exists in different molecular forms (full-length, nitrosylated and cleaved). Yet, attempts to construct assays for the breakdown products of SP-D have not provided additional information regarding COPD [26].

Factors associated with increasing circulating SP-D in healthy subjects include age, BMI and male gender [16,27,28]. Circulating SP-D has been correlated with a variety of pulmonary pathologies [29], and a small comparative study showed that serum SP-D levels could not distinguish COPD from the other pulmonary pathologies [30].

A recent population-based study demonstrated a correlation between serum SP-D and reduced pulmonary function in tobacco smokers [31], suggesting that serum SP-D may serve as a marker for subclinical cigarette smoke induced lung damage. In concordance with this, in a relatively small COPD study, an inverse correlation between serum SP-D and FEV_1_/forced vital capacity (FVC) ratio was found in smokers [22]. This result was however not replicated in the ECLIPSE multi-center cohort study [25,32], nor in a Swedish twin study [33], both of which contained non-smoker control subjects in addition to smoker control subjects.

In the ECLIPSE cohort, higher serum SP-D levels were reported among COPD patients relative to current and former smokers. COPD subjects who had serum SP-D concentrations in the upper 5th percentile of non-smokers had (i) increased risk of exacerbations over the following 12 months [25], and (ii) increased serum SP-D concentrations during exacerbation of COPD compared with stable COPD [34,35]. Data from the ECLIPSE cohort have further demonstrated that baseline serum SP-D levels were associated with a decline in lung density as measured by low-dosage high-resolution computed tomography [36].

### Hard clinical outcomes

Further reports from the ECLIPSE cohort found that baseline serum SP-D levels were associated with 3-year all-cause mortality [37].

### Modification by interventions and correlations between changes in clinical outcomes and biomarker changes

Three cohort studies have showed that treatment with oral corticosteroids resulted in suppressed serum SP-D levels [25,38,39], whereof one showed that serum SP-D correlated with symptom relief and change in FEV_1_% predicted [38].

*In summary*, results from GWAS and animal studies suggest a strong link between SP-D levels and COPD. Smoking highly influences systemic SP-D levels and may be an explanation of the inconclusive findings of association between severity of COPD and systemic SP-D levels. SP-D has so far not proven to be a reliable prognostic tool in advanced COPD; however, stratification for tobacco smoking is warranted in such analyses and has been lacking. Although large studies have found associations between SP-D levels and early loss of pulmonary function as well as mortality and decline in lung density in COPD, these findings need to be further validated and the data representing intervention effects on SP-D are sparse.

### CC-16

CC-16 is predominantly secreted from non-ciliated club cells [22] and is localized to both terminal bronchial epithelia [40] and respiratory bronchiolar epithelia [41,42]. An identical protein, human urinary protein 1 (P1), is secreted in the urogenital tract [43]. Systemic levels of CC-16 correlate well with BALF levels, and appear to be unaffected by the release of P1 from the urogenital tract in healthy subjects [44,45]. Circulating CC-16 levels reflect different chronic pulmonary pathologies, with circulating CC-16 levels being positively associated with severity in sarcoidosis and asbestos-exposed workers [45-49]. However, circulating levels are negatively associated with the severity of asthma [50], cystic fibrosis (CF) [51] and lung cancer [45].

### Biological plausibility

CC-16-deficient mice challenged with hyperoxia showed augmented pulmonary inflammation [52], and *in vitro* studies have indicated that CC-16 inhibits phospholipase A_2_ activity [53] suggesting an anti-inflammatory role of CC-16.

Candidate gene studies have reported an association between a SNP in the *SCGB1A1* gene (Chromosome 11, A38G). Carriers exhibited reduced CC-16 plasma levels and an increased risk of asthma in a child cohort with European ancestry [54]; this association between asthma and SNP A38G was further replicated in adults [55]. However, in a Chinese population, no association between this SNP and COPD was found [56]. In a GWAS where serum CC-16 levels were measured in 1951 COPD subjects, associations between 11 SNPs on chromosome 11 (one of which was located in the *SCGB1A1* gene) and serum CC-16 were found. These 11 SNPs were further evaluated in an additional 2,939 COPD cases and 1,380 smoking controls, and one SNP, located near the *AHNAK* gene, was associated with both COPD risk and frequency of exacerbation during a 2-year follow-up. However, in the same study, this finding could not be replicated in two additional control/COPD populations [18].

### Independent association to disease

Confounding factors that increase systemic levels of CC-16 in healthy subjects include age [57,58], BMI (though with conflicting findings) [33,59], and a decline in glomerular filtration rate [60].

Several studies have shown that circulating levels of CC-16 are lower in healthy smokers relative to non-smokers, while other studies, examining the amount of smoking, have been inconclusive [50,61-63].

In the ECLIPSE study, CC-16 levels were reduced in former and current smokers with COPD relative to current smoking controls [63]. This finding was consistent with findings from previous smaller studies [33,45,64]. When stratifying COPD patients into GOLD groups and by current smoking, lower serum CC-16 levels were seen in current smoking COPD patients relative to former smokers in GOLD2 and GOLD3 but not in GOLD4. In former-smoking COPD patients, a significant inverse correlation was observed between CC-16 and COPD severity. Additionally, CC-16 could distinguish between patients with or without reversibility in former smoking COPD patients [63]. The evaluation of FEV_1_ decline over time in the ECLIPSE cohort showed a weak positive association between serum CC-16 and annual rate of decline in FEV_1_ [2]. This was recently validated in a cohort consisting of 4,724 COPD subjects, where reduced levels of CC-16 were associated with accelerated decline in FEV_1_ over 9 years [65].

Repeated measurements of CC-16 in a sub-population from the ECLIPSE cohort found that serum CC-16 is a stable marker over time [66]. In a study including 357 twins with respiratory symptoms, a positive association with FEV_1_ and an inverse association with residual volume/total lung capacity were found. However, analysis of a sub-cohort of 100 COPD patients within this twin population found no significant association to these measures [33].

### Hard clinical outcomes

Serum CC-16 did not associate independently with mortality in the ECLIPSE cohort [37].

### Modification by interventions and correlations between changes in clinical outcomes and biomarker changes

A pilot randomized clinical trial (RCT) with 16 cachectic COPD patients and 25 controls treated with TNF-α antibody reported a rise in plasma CC-16 in COPD patients after 8 weeks of treatment; treatment effects on other end-points were not considered in this study [67]. In an RCT with approximately 100 subjects in three treatment arms (a p38 mitogen-activated protein kinase (p38 MAPK) inhibitor, salmeterol/fluticasone propionate and placebo), a reduction in CC-16 levels was seen in the salmeterol/fluticasone group after 2 weeks treatment [68].

*In summary*, results from animal studies suggest a causal role of CC-16 in COPD. However, the lack of a strong association between genotype variations, circulating CC-16 levels and risk of COPD indicates that CC-16 associates with pulmonary inflammation in general rather than with COPD pathogenesis explicitly. Furthermore, the inconsistent association to severity in COPD, the lack of association to mortality, the confounding effects in basal variation and the close association between CC-16 and asthma, all make the use of CC-16 as a biomarker in COPD problematic. However, additional studies validating the association between CC-16 and FEV_1_ decline are warranted.

### PARC/CCL-18

PARC/CCL-18 is a chemokine highly expressed in the lungs [69] with a chemotactic effect on primarily lymphocytes [70]. PARC/CCL-18 is synthesized mainly in dendritic and monocytic cells [71], but has also been shown to stimulate fibrinogenic activity and collagen production in lung fibroblasts [72,73]. Due to its predominant production in the lungs, PARC/CCL-18 has been evaluated in multiple pulmonary pathologies. In pulmonary fibrosis, an up-regulation in lung tissue [74] and an association to mortality has been reported [75].

### Biological plausibility

No studies have directly addressed a mechanistic role for PARC/CCL-18 in COPD.

### Independent association to disease

LPS-inhalation challenge in smokers increased levels of PARC/CCL-18 in serum after 24 h [76]. Few studies have evaluated PARC/CCL-18 as a biomarker for COPD. One small study has shown an association between PARC/CCL-18 and FEV_1_, Body Mass Index, Obstruction, Dyspnea score and Exercise capacity (BODE) index, and exacerbation rate [77]; another study reported elevated PARC/CCL-18 during COPD exacerbation [78]. The largest study to date of PARC/CCL-18 in COPD, included different COPD populations: 4,800 subjects from lung health study (LHS) with mild or moderate COPD, 1,800 COPD subjects from the ECLIPSE study representing all GOLD stages, 312 smoking and 226 non-smoking controls, and 89 COPD subjects from a prednisolone intervention study [79]. The results were somewhat contradictory. In LHS, higher PARC/CCL-18 levels associated with lower baseline FEV_1_ and increasing cardiovascular mortality. In the ECLIPSE subjects, PARC/CCL-18 levels were higher in COPD subjects than in controls, but the association with FEV_1_ could not be replicated.

### Hard clinical outcomes

PARC/CCL-18 was associated with all-cause mortality in the ECLIPSE cohort [79].

### Modification by interventions and correlations between changes in clinical outcomes and biomarker changes

Two weeks treatment with prednisolone was associated with a significant reduction in PARC/CCL-18 levels compared to placebo in the ECLIPSE cohort [79].

*In summary*, although some evidence of association between PARC/CCL-18 and severity and mortality in COPD exists, additional validation and data regarding intervention effects on PARC/CCL-18 are warranted.

### CRP

CRP is an acute phase protein, mainly induced by interleukin 6 (IL-6) and is a component of the innate immune response [80]. Increased systemic levels of CRP are seen in a variety of inflammatory conditions, particularly in infections.

### Biological plausibility

The direct link between systemic inflammation and the development and progression of COPD is debated; however, the effect of CRP on activation of the complement system [80] can serve as a factor in maintaining an inflammatory state in stable COPD and thereby contribute to the negative systemic effects associated with COPD.

Although CRP levels are genetically determined [81], SNPs or haplotypes known to effect CRP levels have not been associated COPD risk [18,81-83].

### Independent association to disease

In large population based case–control studies [84], as in large COPD cohorts [84-86] levels of CRP are demonstrably higher in stable COPD patients than in controls after adjusting for the confounding factors: sex, age, tobacco consumption and ischemic heart disease. Reported associations between CRP and airflow limitation have been inconsistent, with reports of weak to moderate unadjusted correlations between FEV_1_ and CRP [87-90]. Analysis of CRP association with COPD severity, after adjustment for age, gender, pack-year history, presence of cardiovascular risk factors or disease and treatment with inhaled corticosteroids, showed an inverse association with 6-minute walking distance (6MWD) [88,91], diffusing capacity (adjusted for age, gender, height, smoking and BMI) [33], and hypoxemia (adjusted for sex, age, body composition and smoking) [87]. However, in one additional study (n = 222) no association with 6MWD or BODE index was found [92].

A link between systemic inflammation and comorbidity in COPD has been suggested, and elevated CRP levels in COPD patients with cardiovascular disease (CVD) [93,94], type II diabetes [94], and lung cancer [94] have been reported. CRP furthermore associated with cardiovascular and cancer mortality [95]. In a recent study, higher levels of CRP were found in a “metabolic” comorbidity cluster with the characteristics of obesity, hyperglycemia, dyslipidemia, hypertension, and atherosclerosis when compared with the cluster of CVD without the metabolic features [96], indicating that the link between COPD, systemic inflammation and CVD could be metabolic impairment. CRP levels are higher during exacerbations than during stable COPD (AUC: 0.73) [78] and can be used to distinguish between exacerbation with or without bacterial infection (AUC: 0.8) [97,98]. In addition, CRP levels were higher in patients with frequent exacerbations compared to stable state patients [85], although CRP levels were not predictive of an exacerbation [99].

CRP has been shown to exhibit high variability over time. For example, in 201 COPD patients with CRP measured at baseline and 3 months later, only 21% had values within 25% of each other [66].

### Hard clinical outcomes

CRP levels have been shown to predict all-cause mortality in a group with mild to moderate disease in a population-based cohort (n = 5000) [95], but not in a smaller (n = 218) cohort with moderate to severe COPD [90]. In the ECLIPSE cohort, higher CRP associated with all-cause mortality but did not alone contribute to better prediction of mortality than a multivariate model [88]. Another large COPD population study found that CRP levels predicted mortality and hospitalization [84].

### Modification by interventions and correlations between changes in clinical outcomes and biomarker changes

In a RCT with 41 COPD subjects, randomized to oral prednisolone, inhaled glucocorticoid or placebo, there was a significant fall in oral prednisolone and inhaled glucocorticoid compared to placebo [100].

*In summary*, evidence that CRP plays a causal role in COPD is controversial. There have been conflicting reports regarding the value of CRP to predict mortality and hospitalizations. Although associations with COPD have been reported, CRP may not be a suitable biomarker in COPD, due to its low specificity and high variability.

### Fibrinogen

Fibrinogen is primarily synthesized in the liver [101], is involved in clotting formation [102] and systemic levels are elevated in an IL-6-stimulated acute phase response [103,104].

### Biological plausibility

The genes coding for three peptide chains in fibrinogen are located on chromosome 4 [105]. Circulating fibrinogen levels are genetically determined [106] and SNPs have been associated with risk of cardiovascular disease [106]. A candidate gene study with selected SNPs in the *FBG* gene encoding fibrinogen did not find an association with circulating fibrinogen levels [107]. A direct role in COPD is not found.

### Independent association to disease

After adjusting for cardiovascular risk factors, several large population based cohorts have found an association between smoking and elevated systemic fibrinogen levels in healthy subjects [108-110]. A meta-analysis from “Fibrinogen Studies Collaboration” consisting of 154,211 apparently healthy subjects from 31 cohorts reported a positive association between fibrinogen and age, female gender, and alcohol abstinence [111].

Fibrinogen is shown to associate with the risk of COPD [112-114]. Higher fibrinogen levels were associated with the rate of decline in FEV_1_/FVC in a population of elderly subjects [115], and in cohorts with stable COPD patients, fibrinogen was shown to associate with FEV_1_ [2], dyspnea, exercise capacity [87] and the composite BODE index [116]. However, a study of 102 COPD patients did not report a significant association of fibrinogen with FEV_1_, but with diffusing capacity of the lung for carbon monoxide (DLCO) [117]. There have further been conflicting reports about the association between fibrinogen and decline in FEV_1_. In a COPD cohort, comprising 148 patients [118] an association between increasing fibrinogen levels and decline in FEV_1_ was found. However, this was not replicated in a small Japanese study (n = 73) [119], nor in the ECLIPSE investigation [2].

Fibrinogen has moreover been evaluated as a tool for distinguishing subgroups of COPD. In a small study of male COPD patients, a group with emphysematous lesions involving more than 15% of the lung parenchyma (n = 24) had higher fibrinogen levels than controls (n = 25) [120]. Elevated levels of fibrinogen in stable COPD patients have been shown to be predictive of risk of exacerbation [99,121,122], and elevated fibrinogen levels have been reported during an exacerbation [121]. Lastly, there has been a trend towards higher fibrinogen levels in patients suffering from exacerbations with accompanying purulent sputum [121], or virus infection [123].

Increased circulating fibrinogen levels have been suggested as a risk factor for cardiovascular disease and associated mortality [124]. In the meta-analysis from “Fibrinogen Studies Collaboration”, a moderately strong association between fibrinogen and risk of coronary heart disease was reported [111]. However, a recent study from same group reported that cardiovascular event risk prediction when adding fibrinogen to a model with established risk factors gave little improvement [125]. In a Danish cohort of 8,656 COPD patients, fibrinogen alone was not a strong predictor for ischemic heart disease or myocardial infarction; however, together with CRP and leukocyte count, and hazard ratios for ischemic heart disease, myocardial infarction and heart failure were approximately two-fold higher in the group with high levels of the biomarkers compared to those with low-levels [94]. On the other hand, a small study of 60 COPD patients along with 20 smoking and 20 non-smoking controls showed no independent association between fibrinogen and flow-mediated vasodilatation (an independent predictor of cardiovascular morbidity and mortality) [38].

### Hard clinical outcomes

Fibrinogen has been shown to associate with both the risk of COPD and of hospitalizations in a Swedish population cohort (n = 5,247) [112], in a Danish population cohort (n = 8,955), which also showed an inverse association to decline in FEV_1_ [113], and in a US population cohort (n = 20,192), that also found associations with GOLD group and mortality [114]. Fibrinogen has further been shown to predict all-cause mortality in COPD [37,126], although in a later investigation, when comparing c-statistics, fibrinogen alone did not contribute to a better prediction of mortality than a basic model (including age, BODE index and hospitalizations).

### Modification by interventions and correlations between changes in clinical outcomes and biomarker changes

In a small study of patients with exacerbation (n = 30), fibrinogen was suppressed by systemic corticosteroid treatment [127]. However, in a study of stable COPD, plasma fibrinogen was not affected by oral prednisolone [25]. In an RCT with approximately 100 subjects in three treatment arms (p38 mitogen-activated protein kinase (p38-MAPK) inhibitor, salmeterol/fluticasone propionate and placebo), treatment with a p38-MAPK inhibitor resulted in an 11% reduction in plasma fibrinogen levels [68].

*In summary*, there has been no emerging evidence of direct causality between circulating fibrinogen levels and COPD. Fibrinogen levels have nevertheless been shown to be independently associated with COPD, prediction of all-cause mortality, and risk of exacerbation. In addition, fibrinogen levels are modifiable by treatment interventions. As a systemic inflammatory marker with less variability over time than CRP, fibrinogen is an interesting candidate biomarker with putative value in distinguishing subtypes and comorbidity clusters in COPD and is currently being taken forward by the collaborative COPD Biomarker Qualification Consortium [128]. However, further investigations of fibrinogen in the assessment of treatment response are still needed.

## Summary and conclusion

A summary of conclusions is provided in Table 1.

**Table 1:** **Summary of evidence of reviewed biomarkers in relation to questions raised by Sin & Vestbo** [4]

	**SP-D**	**CC-16**	**PARC/ CCL-18**	**CRP**	**Fibrinogen**
Is there a strong biological plausibility in terms of its role in pathogenesis of disease?	Evidence from animal studies and gene-association studies [10-19]	Suggested from *in vitro*, animal study and gene-association studies [18,52,53]	N.A.	Experimental data suggest role in systemic effects and comorbidity [81]	Experimental data suggest role in systemic effects and comorbidity [108,109]
Is there a strong, consistent and independent association between the biomarker and COPD?	Level IIb[16,20-35]	Level IIb [33,45,63-66]	Level III [77-79]	Conflicting results from large population studies [78,85-99]	Level IIb [32,87,112-122,125]
Is there a strong, independent association between the biomarker and hard clinical outcomes such as mortality and hospitalisations?	Level IIa [36,37]	No evidence [37]	Level IIb [79]	Level IIa; on all cause mortality [84,86,99]	Level IIa [37,112-114,126]
Is there evidence from randomised controlled trials that the biomarker is modifiable by interventions?	Evidence from 3 cohort studies of prednisolone treatment [25,38,39]	Evidence from one RCT of TNF-R antibody treatment [67] and one RCT in salmeterol/fluticasone propionate-arm [68]	Evidence from one RCT with prednisolone treatment [79]	Evidence from one RCT with inhaled glucocorticoid, prednisolone or placebo [100]	Evidence from one RCT with p38 MAPK inhibitor[68,127]
Is there evidence from randomised controlled trials that changes in the biomarker status results in changes in an important (and accepted) clinical outcome (e.g. mortality, exacerbations, rate of decline in FEV_1_ and health status)?	N.A.	N.A.	N.A.	N.A.	N.A.

Ia - Evidence from Meta-analysis of Randomized Controlled Trial, Ib - Evidence from at least one Randomized Controlled Trial, IIa - Evidence from at least one well designed controlled trial which is not randomized, IIb - Evidence from at least one well designed experimental trial, III - Evidence from case, correlation, and comparative studies, IV - Evidence from a panel of experts. SP-D: Surfactant protein D, CC-16: club cell protein 16, PARC/CCL-18: pulmonary and activation-regulated chemokine 18, CRP: C-reactive protein. N.A.: no available studies.

### Biological plausibility

Findings from animal studies and gene-association studies point at a plausible role of the pulmonary proteins SP-D and CC-16 in COPD.

### Independent association to disease and hard clinical outcomes

There is evidence that SP-D, CC-16 and PARC/CCL-18 reflect disease severity. Findings indicate association between pulmonary inflammatory proteins and risk of exacerbations, hospitalizations and death. However, results do not show the same consistency as with systemic inflammatory proteins. Fibrinogen appears to be a more specific biomarker for COPD than CRP, and is demonstrated to predict exacerbation risk and all-cause mortality. However, systemic inflammatory proteins are influenced by additional pathologies such as heart disease and metabolic disturbances, which are both common comorbidities in COPD. As the only one of the above reviewed biomarkers CC-16 has showed to associate with decline in lung function, which indicates a utility in evaluation of disease activity.

### Modification by interventions and correlations between changes in clinical outcomes and biomarker changes

All of the reviewed biomarkers have showed to be modifiable by either oral prednisolone or inhaled glucocorticoid to different extents. However, only few randomized clinical trials have been evaluating this matter. Though biomarkers in COPD are extensively investigated, most of the studies comprise cohort and experimental studies. Randomized clinical trials evaluating changes in biomarkers in relation to interventions and clinical outcome are lacking.

*In conclusion*, none of the biomarkers in this review fulfill all of the criteria presented by Sin & Vestbo [4] and an additive approach, with different biomarkers combined, may contribute to increased specificity and sensitivity for prognosis of COPD.
